# Diverse Gene Cassettes in Class 1 Integrons of Facultative Oligotrophic Bacteria of River Mahananda, West Bengal, India

**DOI:** 10.1371/journal.pone.0071753

**Published:** 2013-08-09

**Authors:** Ranadhir Chakraborty, Arvind Kumar, Suparna Saha Bhowal, Amit Kumar Mandal, Bipransh Kumar Tiwary, Shriparna Mukherjee

**Affiliations:** Department of Biotechnology, Omics Laboratory, University of North Bengal, Siliguri, West Bengal, India; Beijing Institute of Microbiology and Epidemiology, China

## Abstract

**Background:**

In this study a large random collection (n = 2188) of facultative oligotrophic bacteria, from 90 water samples gathered in three consecutive years (2007–2009) from three different sampling sites of River Mahananda in Siliguri, West Bengal, India, were investigated for the presence of class 1 integrons and sequences of the amplification products.

**Methodology/Principal Findings:**

Replica plating method was employed for determining the antibiotic resistance profile of the randomly assorted facultative oligotrophic isolates. Genomic DNA from each isolate was analyzed by PCR for the presence of class 1 integron. Amplicons were cloned and sequenced. Numerical taxonomy and 16S rRNA gene sequence analyses were done to ascertain putative genera of the class 1 integron bearing isolates. Out of 2188 isolates, 1667 (76.19%) were antibiotic-resistant comprising of both single-antibiotic resistance (SAR) and multiple-antibiotic resistant (MAR), and 521 (23.81%) were sensitive to all twelve different antibiotics used in this study. Ninety out of 2188 isolates produced amplicon(s) of varying sizes from 0.15 to 3.45 KB. Chi-square (χ^2^) test revealed that the possession of class 1 integron in sensitive, SAR and MAR is not equally probable at the 1% level of significance. Diverse antibiotic-resistance gene cassettes, *aadA1, aadA2, aadA4, aadA5, dfrA1, dfrA5, dfrA7, dfrA12, dfrA16, dfrA17, dfrA28, dfrA30, dfr-IIe, blaIMP-9, aacA4, Ac-6′-Ib, oxa1, oxa10* and *arr2* were detected in 64 isolates. The novel cassettes encoding proteins unrelated to any known antibiotic resistance gene function were identified in 26 isolates. Antibiotic-sensitive isolates have a greater propensity to carry gene cassettes unrelated to known antibiotic-resistance genes. The integron-positive isolates under the class *Betaproteobacteria* comprised of only two genera, *Comamonas* and *Acidovorax* of family *Comamonadaceae,* while isolates under class *Gammaproteobacteria* fell under the families, *Moraxellaceae*, *Pseudomonadaceae*, *Aeromonadaceae* and *Enterobacteriaceae*.

**Conclusions:**

Oligotrophic bacteria are good sources of novel genes as well as potential reservoirs of antibiotic resistance gene casettes.

## Introduction

Bacteria that grow and multiply using low concentration of organic substrates are oligotrophs. Oligotrophic bacteria can be broadly classified into two groups, obligate and facultative. Obligate oligotrophic bacteria grow only in nutrient-poor media and fail to grow in nutrient-rich media, while facultative oligotrophic bacteria have the capacity to grow in both nutrient-poor and nutrient-rich media. A nutrient-poor medium, R2A, was formulated for cultivating environmental bacteria (oligotrophic) from potable water [1]. It was found that bacterial counts on R2A agar were 34.3% greater than the bacterial counts on standard plate count agar [2]. Several oligotrophic bacteria from different natural sources were studied for their substrate requirement [3]. Oligotrophic bacteria from an estuarine environment showed that 90% bacteria belonged to the known genera, *Alcaligenes, Corynebacterium, Hyphomicrobium, Hyphomonas, Listeria, Nocardia, Pedomicrobium, Planococcus, Sphaerotilus, Streptothrix,* and *Streptomyces;* and the remaining 10% were unidentified sheathed bacteria [4]. Oligotrophic bacteria isolated from hospital tap-water revealed the presence of 23.6% *Methylobacterium,* 13.2% *Pseudomonas* but the rest could not be identified [5]. Such bacteria have also been reported from rhizospheres of various soil samples [6]. Some workers have used 100–10,000 fold diluted nutrient agar for isolation and cultivation of oligotrophic bacteria from different sources [7], [8]. An earlier report showed that on plating environmental samples, more colonies representing diverse bacterial communities were evident on diluted Luria Bertani (LB) than undiluted LB broth supplemented with agar [9]. Hence, diluted LB or diluted Luria broth supplemented with agar was used for isolation and enumeration of oligotrophic bacteria [10]–[12]. Oligotrophic strains like *Acinetobacter johnsonii* MB52, *Klebsiella pneumoniae* strain MB45, and *Brevibacterium siliguriense* from river water were isolated on diluted Luria agar [11], [12], [13].

Although studies on antibiotic-resistance including methodology and standard cutoff limits for determining susceptibility/resistance were largely confined to copiotrophic bacteria, even so there are a few reports on antibiotic resistance in oligotrophic bacteria [7], [12], [14]–[17]. Multiple-antibiotic-resistant (MAR) strains (resisting up to 14 different antibiotics) have also been reported from the pristine Lechuguilla Cave [18]. Recently, two large plasmids, pREV1 and pREV2 (about 150 and 250 KB, respectively), isolated from an oligotrophic bacterium, *Ancylobacter vacuolatus*, were shown to carry resistance genes for chloramphenicol and trimethoprim in addition to genes coding functions related to oligotrophy [19]. Oligotrophic bacteria therefore can be viewed as the potential reservoir of antibiotic resistance genes, and such genes can be disseminated to pathogens through gene swapping in the environment. Integrons, widely present in bacteria, are the dynamic platforms for acquiring and spreading gene cassettes which often bear antibiotic-resistance gene. The present work was undertaken to reveal the incidence of class 1 integrons in oligotrophic bacteria of a city-waste polluted river of northern West Bengal, Siliguri, India, with special emphasis on predicting functions of the integron-borne gene cassettes. This investigation has expanded the horizon to swot integron-borne gene cassettes for novel genes.

## Materials and Methods

### Ethics Statement

This study did not require any specific permission for describing the locations of the sampling stations on River Mahananda; as such this was the part of the research project funded by the Department of Biotechnology (DBT), Government of India. Moreover, the field studies did not involve any endangered or protected species.

### Selection of Sampling Sites, Sampling Strategy and Collection of Samples

**Figure 1:**
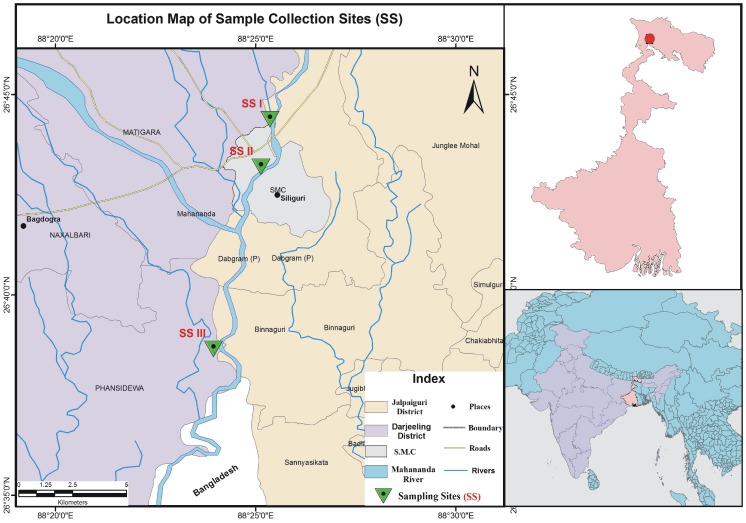
Geographical Map showing sampling sites (SS). The map was constructed using ArcGIS software. SSI, Champasari; SSII, Mahananda Bridge at Siliguri; and SSIII, Fulbari.

Three sampling sites on River Mahananda were selected after examining the topographic map of the river. The sampling sites were: SS I (upstream, the entry point of the river into Siliguri city) – near Champasari (26°44′22.76″N, 88°25′22.41″E); SS II (midstream, the midpoint of the river at Siliguri city) – under the Mahananda bridge (26°43′11.52″N, 88°25′8.80″E) situated in the middle of the city; and SS III (downstream, exit point where the river leaves the main township) – near the dam at Fulbari (26°38′38.89″N, 88°23′57.78″E) (Fig. 1). Three grab samples from each sampling site were collected and studied following standard methodology (APHA 1989) in every month except July and August (monsoon months). Ninety composite water samples (a mixture of three grab samples) [3 sampling sites × 10 composite samples per year per sampling site × 3 years] were analyzed from three sampling sites in a span of three consecutive years (2007–2009).

### Enumeration of Oligotrophic Bacteria on Nutrient Poor Agar (NPA) Medium

0.1 ml of water sample [undiluted and diluted water (10^−1^, 10^−3^, 10^−5^, 10^−7^, and 10^−9^)] was spread uniformly on NPA (nutrient poor agar; composition in g/L: 0.01, peptone; 0.005, yeast extract; 0.005, sodium chloride; 15, agar, pH 7.2) and plates were incubated at 30°C for 72 h. Total culturable oligotrophic bacterial load of each water sample was quantified by counting bacterial colonies [Colony Forming Unit (CFU/ml)] on NPA medium.

### Screening of Facultative Oligotrophic Bacteria from the Oligotrophic Bacterial Population

Discrete colonies evident on NPA plates (plated with 0.1 ml water samples of different dilutions) were numbered serially (1, 2, 3…n). Approximately, 100±10 random numbers (out of >300 discrete colonies) were generated using a research Randomizer tool (www.researchrandomizer.com). Colonies corresponding to the random numbers were then picked up with the help of sterile loop and transferred to respective grids of the first generation master plate made of R2A agar (HiMedia ME962). The first generation master plates were then replicated separately on NRA (nutrient rich agar; composed in g/L: peptone, 10; yeas, 5; sodium chloride, 5; agar, 15), NPA and R2A agar plates and further incubated at 30°C for 72 h. Isolates showing growth on NRA, NPA and R2A agar plates were considered as facultative oligotrophic bacteria while colonies showing no growth on NRA but significant growth on NPA and R2A agar plates were considered as obligate oligotrophic bacteria. The obligate oligotrophic bacteria were not included in this study due to its slow growth and ambiguity in forming colonies. Facultative oligotrophic bacterial colonies identified from the master plates were further remembered and randomized to obtain 25±1 random numbers. The colonies corresponding to each of the 25±1 random numbers were diluted streaked onto R2A agar plate to obtain pure cultures of the isolates. The second generation master plate was prepared on R2A agar with pure cultures of facultatively oligotrophic isolates. In this way, a total of 2188 facultative oligotrophic isolates irrespective of the knowledge of the phenotype (sensitive, single or MAR) of the second generation master plate were selected for the further studies.

### Determination of Antibiotic Susceptibility/Resistance of Facultative Oligotrophic Bacteria

Replica plating method was employed for determining the antibiotic resistance profile of individual isolates. The second generation master plate, prepared with the pure cultures of facultative oligotrophic isolates, was replica plated onto the R2A plate containing antibiotic of defined concentration (specific for each antibiotic) as described previously [11]. Antibiotic Resistance index (mentioned throughout as RI) for each test isolate was calculated by dividing the number of antibiotics to which the isolate was resisted by the number of antibiotics exposed.

### Amplification, Cloning and Characterization of the Variable Region of Class 1 Integron

All facultative oligotrophic isolates (total number: 2188) were examined for the presence of class 1 integrons, using a highly reproducible PCR strategy described earlier [20]. PCR reaction was performed on BIO-RAD DNA engine (Peltier Thermal cycler). In all reactions, PCR set up containing the whole cell DNA of *Morgnella* Sp. TR90 (class 1 integron-bearing bacterium) was used as positive control. The genomic DNA of *Escherichia coli* JM109 (devoid of class 1 integron) and sterile distilled water were used as negative controls. The amplicons were cloned and sequenced according to the method described previously [11]. Sequence analyses were done with the available Bioinformatics tools (Vec screen, ORF finder, BlastN and BlastP suite, Conserved Domain search, ProtParam analyses, TMMOD tool, Sequence Alignment tools, Ident and Sim). The experimental design has been summarized in Fig. S1.

### Provisional Genus Identification of Isolates Bearing Class 1 Integron

The putative genera of the isolates possessing class 1 integrons were primarily determined by using numerical taxonomy. The phenotypic data were converted into binary characters (1, for positive character and 0, for negative character) and similarity matrix was generated by using the Sneath and Sokal methodology [21]. The Jaccard (Tanimoto) coefficient was computed from the set of variables (similarity: a/a+b, where a, is the homolog character present in two bacterial isolate and b is the number of non-homolog character present in two bacterial isolate). The similarity dendogram was generated using unweighted pair group method (UPGMA) by the help of DendroUPGMA tool available at http://genomes.urv.cat/UPGMA/. The isolates, clustered together in groups, were processed for total protein profiling. In order to extract total proteins, 3–4 colonies of each isolate were suspended in 0.2 ml of 2X SDS-gel loading buffer and list at 100°C separately. The supernatant (cell lysate) containing total cellular proteins were separated using 12% SDS-PAGE; and bands were visualized under white light after coomassie blue staining [22]. Separated protein bands per lane were compared and considered identical when all the protein bands (migrated bands) in lanes were at the same distances. One representative culture of each group of bacteria, exhibiting a similar phenotype and nearly identical protein band pattern, was used for amplification of 16S rRNA gene. Whole cell DNA extraction, cloning and sequencing were done as described previously [12].

### Statistical Test

The experimental data were coded, scored and analyzed using SPSS software. The contingency analysis was used to test significance between the different phenomena on the basis of classification of attributes by applying the Chi-square (χ^2^) test. Levels of significance were obtained at the p-values <0.01, <0.05 and >0.05. The p-value of <0.01 was regarded as highly significant.

## Results

### Occurrence of Sensitive and Antibiotic-resistant (SAR and MAR) Facultative Oligotrophic Bacteria of River Mahananda

**Figure 2:**
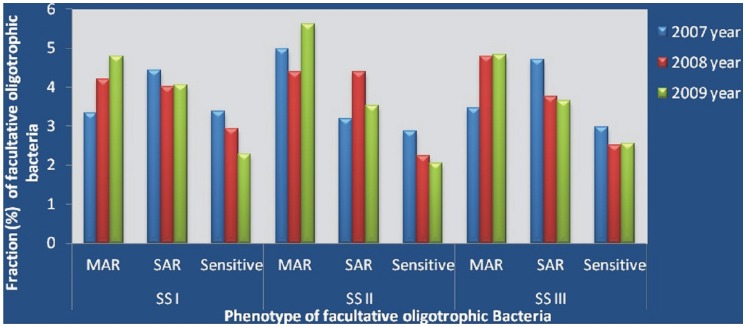
Comparative analyses of MAR, SAR and sensitive facultative oligotrophs. Water samples were collected from three different sampling sites (SS) from the River Mahananda in three consecutive years (2007–2009).

Oligotrophic bacterial density in water samples of the River Mahananda [from three sampling sites (SS I, upstream; SS II, midstream; SS III, downstream; at Siliguri) for three successive years] ranged from 1×10^3^ to 5.9×10^4 ^CFU/ml. The percentage occurrence of MAR facultative oligotrophic bacteria was found to be increasing in water samples of a SS I and SS III, while such consistent increase was not observed with SS II samples. The occurrence of SAR bacteria was found to be decreased from 2007 to 2009 at SS III. Frequencies of SAR bacteria at SS I remained more or less constant during the year 2008 and 2009. A gradual decrease in sensitive and concomitant increase in MAR bacteria was observed from 2007–2009 in water samples of SS I and SS III. However, MAR bacteria were more frequent than sensitive or SAR isolates at SS II with a gradual decrease in sensitive isolate (Fig. 2). From the pool of total oligotrophic (both obligate and facultative) bacteria, facultative ones were selected by a replica plate method for further study. A total of 2188 facultative isolates were randomly selected for determination of susceptibility/resistance towards 12 different antibiotics tested. Of them, 76.2% (1667) were antibiotic-resistant and 23.8% (521) were sensitive to all antibiotics. Amongst antibiotic-resistant isolates, 47% (782) exhibited resistance to single antibiotic, designated as SAR and 53% (885) were resistant to two or more than two antibiotics, designated as MAR. Among the MAR group, 19.86% (331) were resistant to two, 11.64% (194) to three, 6.54% (109) to four, 4.67% (78) to five, 2.46% (41) to six, 3.78% (63) to seven, 1.62% (27) to eight, 1.02% (17) to nine, 0.78% (13) to ten, and 0.48% (8) to eleven antibiotics. Only 0.24% (4) of the selected isolates were resistant to all the 12 antibiotics tested.

### Detection of Class 1 Integrons in Facultative Oligotrophic Isolates and Determination of Antibiotic-resistance-profile (ARP) of the Integron Positive Isolates

Class 1 integrons were detected in 90 (4.1%) out of 2188 isolates. To detect inserted gene cassettes, variable regions of class 1 integrons were amplified with primers 5′ CS and 3′ CS, which are complementary to 5′ and 3′ conserved segments flanking the inserted DNA [20]. The size of the amplicons varied from 0.15 to 3.45 KB (Table 1,2). Sequence analyses showed that most of the amplicons of size >0.7 kB carried antibiotic resistance genes in their gene cassettes. Amplicons varying between 0.1 to 0.7 kB were found to be either empty class 1 integron or contained sequences coding for hypothetical proteins. The size of the amplicons ranging from >0.1 to <0.5 KB, >0.5 KB to <0.7 KB, >0.7 to <1.0 KB, ≥1. 0 to ≤1. 5 KB, and >1.5 to ≤2.0 were detected in six, eleven, sixteen, thirty three, and twenty three isolates respectively. An amplicon of ∼3.5 KB amplicon was recorded in MB35. A very short variable region of 153 bp was amplified in two strains, MB62 and MB63, while 223 bp amplicon was generated from isolates MB05 and MB70. The amplicon of size ∼1.0 kb was predominating and was detected in 24.4% (22/90) of the total integron positive isolates. Two types of open reading frames, (i) bearing no homology with the described sequences coding for resistance to any known antibiotics (Table 1), and (ii) sequences producing significant homology with already existing antibiotic resistance genes (Table 2), were noted. On the basis of RI, the isolates could be categorized into 11 groups as shown in Table S1. Maximum and minimum incidence of integrons was observed in isolates falling in 0.66 and 0.08 RI group respectively (Fig. S2). Of the 90 class 1 integron bearing isolates, 18 (22%) were sensitive to all the twelve antibiotics, 07 (7.8%) were SAR and 65 (72.2%) were MAR. Among 65 MAR isolates bearing class 1 integrons, 7 (10.77%) were resistant to two, 8 (12.3%) to three, 5 (7.7%) to four, 9 (13.84%) to five, 12 (18.46%) to six, 9 (13.84%) to seven, 10 (15.4%) to eight, 1 (1.53%) to nine, 3 (4.6%) to ten, 1 (1.53%) to eleven antibiotics tested. The facultative oligotrophic bacteria resistant to all twelve antibiotics used in this study did not yield any amplicon of class 1 integron. The antibiotics-resistance-profile of the ninety class 1 integron bearing facultative oligotrophic bacteria are shown in Table S1. On assuming the data agree with the null hypothesis that class 1 integrons is equally probable (1∶1: 1) in the three classes (sensitive, SAR, and MAR), the observed frequencies of the three classes were compared with the expected frequencies by the test statistic χ^2^. Since the observed value of χ^2^ (viz. 63.26) is greater than the tabulated value 9.21 at 1% for 2 degrees of freedom, we can reject the null hypothesis at 1% level of significance and conclude that the possession of class 1 integron in sensitive, SAR, and MAR is not equally probable.

**Table 1:** Isolates bearing gene cassettes unrelated to antibiotic resistance.

RI^a^	Isolate	Approx length (bp)	Nature of gene cassette	Cassette encoded Function	Ac. No.
0.0	OB 12	1000	*orf1*	Hypothetical protein	AM997272
	MB 05	223	*mts*	Ribosomal methyl transferase	AM991331
	MB 08	608	*orf1*	Hypothetical protein	FN178516
	MB 12	1000	*livM1*	Branched chain ABCtransporter	AM997273
	MB 16	1000	*appA*	Oligopeptide ABC transporter	AM991327
	MB 22	1010	*fgam*	Phosphoribosyl formylglycinamide synthase	AM991334
	MB 44	522	*orf1*	Hypothetical protein	MB41^b^
	MB 48	1095	*orf1*	Hypothetical protein	FN178520
	MB 54	527	*orf1*	Hypothetical protein	FM955254
	MB 55	959	*orf55, fgam*	Hypothetical protein, Phosphoribosylformyl glycinamide synthase	HE653232
	MB 56	900	*fhaC*	Putative hemolysinactivator protein	FM955483
	MB 58	624	*tnp*	Transposase, IS4family protein	FM955255
	MB 71	627	*tnp*	Transposase, IS4family protein	MB58^b^
	MB 83	435	*rsu*	Ribosomal large subunit pseudouridine synthase B	OB05^b^
0.08	OB 05	489	*rsu*	Ribosomal large subunit pseudouridine synthase B	AM997271
	MB 09	682	*hsdR*	DNA degradation	AM991332
	MR 01	951	*fgam*	Phosphoribosyl formylglycinamide synthase	FN561626
0.16	MB 51	794	*orf 1*	Hypothetical protein	AM997281
	MR 02	513	GGDEF	Signal transduction	FN561627
0.33	MB 81	522	*orf1*	Hypothetical protein	MB 41^b^
	MR 04	1300	SNF2 family	ATP-dependent helicase	FN561629
0.41	MB 80	522	*orf1*	Hypothetical protein	MB41^b^
0.50	MB 41	522	*orf1*	Hypothetical protein	**HE653229**
0.58	MB 28	1620	*ydcR*	Bifunctional putativeTranscriptional regulator	FM179326
	MB 19	1500	*yfkc*	Putative reversetranscriptase maturase	AM997282
	MB 70	223	*mts*	Ribosomal methyl transferase	MB05^b^

a Resistance index;

b gene sequence identical to that isolate.

**Table 2:** Isolates bearing gene cassettes related to antibiotic resistance or bearing no cassettes (empty class 1 integron).

RI^a^	Isolate	Approx length (bp)	Nature of gene cassette	Cassette encoded Function	Ac. No.
0.0	MB 39	1009	*aadA2*	STR	AM997277
	MB 43	792	*aacA4*	KAN	HE653231
	MB 46	1009	*aadA2*	STR	AM997280
	MB 63	153	*In*0	Empty class 1 integron	FM958478
0.08	MB 20	1009	*aadA1*	STR	MB 32b
	MB 24	1003	*aadA1*	STR	AM991326
	MB 50	1009	aadA1	STR	MB 32b
	MB 57AMB 57B	1242 (i)973 (ii)	*dfrA1-orfB* (i)*aadA1* (ii)	TMP-Hypothetical (i)STR (ii)	HE653233 (i)HE653234 (ii)
0.16	MB 23	1009	*aadA1*	STR/SPEC	MB 32b
	MB 36	1009	*aadA1*	STR/SPEC	AM937245
	MB 49	792	*aacA4*	KAN	MB43b
	MB 52	1694	*dfrA28- aadA1*	TMP-STR	FN263373
	MB 64	769	*dfrA7*	TMP	MB31b
0.25	MB 03	1647	*dfrA28-aadA1*	TMP-STR	AM937241
	MB 21	1569	*dfrA1- aadA1*	TMP-STR	AM937243
	MB 26	1571	*dfrA1-aadA1*	TMP-STR	AM991328
	MB 32	1009	*aadA1*	STR/SPEC	AM991330
	MB 33	1009	*aadA1*	STR/SPEC	AM991333
	MB 42	862	*aacA4*	KAN	HE653230
	MB 67	1913	*dfrA12-orf40A- aadA2*	TMP-Hypothetical-STR	MB40Ab
	MB 77	730	*dfrA30*	TMP	MB72b
0.33	MR 03	737	*dfrA16*	TMP	FN561628
	MB 47	1569	*dfrA1- aadA1*	TMP-STR	FM179327
	MB 62	153	*In*0	Empty class 1 integron	FM998811
0.41	MB 18	1048	*aadA5*	STR/SPE	AM937242
	MB 31	769	*dfrA7*	TMP	HE650981
	MB 40AMB 40B	1913 (i)704 (ii)	*dfrA12- orf40A-aadA2* (i)*yrf1* (ii)	TMP-Hypothetical-STR(i)Helicase (ii)	FM179328 (i)AM997278 (ii)
	MB 59	1543	*dfrA1-aadA1*	TMP-STR	HE653235
	MB 66	1009	*aadA1*	STR/SPE	HE650982
	MB 72	730	*dfrA30*	TMP	HE650983
	MB 74	1242	*dfrA1-orf38*	TMP-Hypothetical	MB38b
	MB 75	1242	*dfrA1-orf38*	TMP-Hypothetical	MB38b
0.5	MB 27	1614	*dfrA17-aadA5*	TMP-STR	AM937244
	MB 30	1664	*dfrA7-aadA5*	TMP-STR	HE650980
	MB 60	1556	*dfrA1-aadA1*	TMP-STR	HE653236
	MB 61	1913	*dfrA12-orf40A-aadA2*	TMP-Hypothetical-STR	MB40Ab
	MB 69	1543	*dfrA1-aadA1*	TMP-STR	MB59b
	MB 78	1694	*dfrA28-aadA1*	TMP-STR	MB52b
	MB 82	2013	*Oxa1-aadA1*	Bla-STR	HE650986
	SR 19	729	*dfrA5*	TMP	FN396373
	OD 05	1009	*aadA1*	STR	FN396375
	OD 08	1009	*aadA1*	STR	FN396376
	OC 16	1664	*dfrA17-aadA5*	TMP-STR	FN396368
0.58	MB 29	1657	*dfrA7-aadA5*	TMP-STR	HE650979
	MB 34B	1663	*dfrA17-aadA4*	TMP-STR	AM997275
	MB 37A	1661	*dfrA17-aadA5*	TMP-STR	AM991329
	MB 53	1664	*dfrA17-aadA5*	TMP-STR	FM179325
	MB 73	1664	*dfrA7-aadA5*	TMP-STR	bMB30
	OC 78	1170	*aac-6′-Ib*	STR	FN396372
0.66	MB 25	1606	*dfrA17-aadA4*	TMP-STR	AM997274
	MB 35	3454	*blaIMP-9-aacA4-oxa-10-aadA2*	Kan-Bla-STR	FN178517
	MB 45	667	*dfrA30*	TMP	AM997279
	MB 65	1556	*dfrA1-aadA1*	TMP-STR	MB60b
	MB 76	769	*dfrA7*	TMP	MB31b
	MB 79AMB 79B	1167 (i)1009 (ii)	*dfrIIe-arr2* (i)*aadA1* (ii)	TMP-RMP (i)STR (ii)	HE650984 (i)HE650985 (ii)
	OD 10	1350	*aadA*	STR	FN396374
	OC 24	1521	*dfrA17-aadA5*	TMP-STR	FN396369
	OD 21	1009	*aadA2*	STR	FN396378
	OD 24	1000	*dfrA12*	TMP	FN396377
0.75	OC 74	1000	*aac-6′-Ib*	STR	FN396370
0.83	MB 38	1241	*dfrA1-orf38*	TMP-Hypothetical	AM997276
	MB 68	1543	*dfrA1-aadA1*	TMP-STR	MB59b
	OC 75	1400	*dfrA17*	TMP	FN396371
0.91	NV 66	1009	*aadA2*	STR	FN396367

a Resistance index (RI); ^b^ gene sequence identical to that isolate.

TMP, trimethoprim; STR, streptomycin; KAN, kanamycin; SPEC, spectinomycin.

### Sequence Characterization of the Gene Cassettes Bearing No Homology to Any Known Antibiotic Resistance Gene

The cassettes, containing open reading frames (ORFs) for which no homology could be found to any known antibiotic resistance gene in sequence databases, were identified in 26 (∼29%) of the total 90 isolates. The features of such gene cassettes were analyzed and compared with the sequences existing databases (Table 1). The sequences present in the gene cassettes of OB05 and MB83 were found 75% identical (nucleotide-nucleotide) to the part of the genome of *Acidovorax avenae* subsp. *citrulli* (Ac. No. CP000512). The feature present in that part of *Acidovorax* genome (bearing resemblance with gene cassette) included a portion of ribosomal large subunit pseudouridine synthase B. Sequence analysis of the gene cassettes of MB05 and MB70 revealed the presence of a ORF that encoded a polypeptide of 64 amino acids; the translated protein showed 73% identity with the methyl transferase of *Ralstonia eutropha* (Ac no. AAZ62060). A translated polypeptide from the gene cassette sequence of the bacterium MB09, signifying type I site-specific deoxyribonuclease, HsdR family, exhibited nearly 42% identity with a protein of the same family, found in *Thiomicrospira crunogena* (Ac. No. YP390604). The gene cassette of MB12 showed the presence of a single ORF (*livM1*) which codes for 154 amino acids long polypeptide. BlastP analysis of this putative polypeptide exhibited 72% identity with ABC transporter permease of the bacterium *Aromatoleum aromaticum* EbN1 (Ac. No. NC_006513). Further analysis has revealed the presence of four transmembrane regions in the predicted LiVM1 protein of MB12 (Protein ID: CAQ53856). The predicted transmembrane (TM) regions were found at amino acid position 2–20, 50–74, 87–110, and 119–138 in ABC transporter protein of the bacterium MB12. The conserved domain (CDD: conserved domain database tool available at www.ncbi.nlm.nih.gov) search for a putative translated product revealed to be one of the members of two TM subunits which play role in the uptake of branched chain amino acids. The theoretical pI and instability index of the putative ABC transporter was computed to be 9.3 and 19.13 respectively. The protein was predicted as a stable protein with an estimated half life of 10 h (*in vivo* with respect to *E. coli*) with a high aliphatic index of 127.21. MB16 was found to carry gene cassette carrying *appA* gene encoding bacterial extracellular solute binding protein. ProtParam computing showed that the extracellular binding protein was stable and its instability index was computed to be 18.94. The GRAVY (grand average hydropathicity), aliphatic index and theoretical pI were computed to be −0.087, 78.5, and 9.26 respectively. Psortb, a tool for subcellular localization prediction showed that the translated product of *appA* gene was periplasmic with localization score of 9.44. Sequence analysis of the gene cassette obtained from MB 19 has shown the presence of a unique hybrid DNA sequence. In the 867 nucleotide long sequence, a continuous stretch of 242 nucleotides (from 3 to 244) produced 96% identity with a vertebrate (*Lepilemur dorsalis)* genomic fragment (Ac. No. AJ244007) and residual 623 nucleotide stretch (from nucleotides 245 to 867) produced 74% identities with *Burkholderia xenovorans* LB400 genomic DNA. This genomic DNA region of *B. xenovorans* codes for the putative reverse transcriptase maturase protein. MB 19 gene cassette contained an ORF coding for 227 amino acids long polypeptide. This translated polypeptide showed 65% identity with reverse transcriptase maturase of *Burkholderia cenocepacia* HI2424 (Ac. No. YP833935). An ORF of MB22 gene cassette encoded for a putative protein of 164 amino acid which shared 81% amino acid identity with phosphoribosylformylglycinamidine synthase (FGAM synthase) of *Acinetobacter baumannii* (Ac. No. YP001712860). The partial sequence of the amplicon from MB 28 (408 nucleotide), found 86% identical with *Klebsiella pneumoniae* subsp. *pneumoniae* MGH 78578 (Ac. No.CP000647) genomic DNA, was predicted to code for a bifunctional putative transcriptional regulator protein. This truncated protein of 84 amino acid shared homology by 96.3% with the similar protein present in *Klebsiella pneumoniae* (Ac. No.CP000647). The complete gene cassette sequence of 704 bp of MB 40B (Table 2) yielded no significant homology with any nucleotide sequence available in the database. The same sequence yielded a complete ORF of 102 amino acids and BlastP analysis of this protein sequence revealed 58% identity with helicase domain protein of *Verminephrobacter eiseniae* EF01 (Ac. No. ABM585806). The putative proteins translated from gene cassettes of OB 12, MB 08, MB41, MB 44, MB 48, MB 51, MB 54, MB55, MB80 and MB81 did not produce any significant homology with any protein sequences available in the database. All of them were characterized as hypothetical proteins. All these polypeptides shared very low level of identity (2.03 to 25.19%) among each other. The protein derived from ORF of MB54 gene cassette exhibited a conserved domain belonging to the UPF0153 superfamily. Protein homology and CDD search of a putative polypeptide of 178 amino acid residues derived from ORF of MB 56 gene cassette was found to encode hemolysin activator/secretion protein. This putative hemolysin activator protein of MB56 produced 55% identity with the hemolysin activator protein of *Acinetobacter* sp. (Ac. No. YP045656). Such proteins are involved in intracellular trafficking and secretion. The gene cassettes of MB 58 and MB71 yielded a truncated ORF of 181 amino acids. The BlastP analysis of the said truncated polypeptide yielded best score (97% identity) with transposase protein of *Acidovorax* sp. (Ac. No. YP987142).

### Sequence Characterization of the Gene Cassettes Bearing Significant Homology to Known Antibiotic Resistance Gene

About 71% (64/90) gene cassettes were found to carry antibiotic-resistance genes (Table 2). The most common carriages were aminoglycoside adenyltransferase gene cassettes such as *aadA, aadA1, aadA2, aadA4,* and *aadA5* conferring resistance to streptomycin/spectinomycin antibiotics. Two types of dihydrofolate reductases, type-A (*dfrA1, dfrA5, dfrA7, dfrA12, dfrA16, dfrA17,* two novel *dfrA* genes, *dfrA28* and *dfrA3*) and type-B (*dfr-IIe*), conferring resistance to trimethoprim, were found. The gene *dfrA28* (Ac. No. FN263373) was reported to be novel having 519 bp long ORF with shared homology (identity) of 76.4% at the amino acid level to the *dfrA1* of *E. coli* (Ac. No. AJ419168) [11]. Aminoglycoside acetyltransferase gene cassette (*aac*-6′-*Ib*) was detected in only two isolates. The *aacA4* gene cassettes conferring resistance to kanamycin was detected in four isolates.

Thirty-three isolates carried single antibiotic-resistance gene cassette in their Class 1 integrons. Single gene cassette, *aadA1*, and *aadA2*, was present in nine and four isolates respectively while *aadA5* gene cassette was found only in one isolate, MB18. A single gene cassette, *dfrA30*, was observed in three isolates, MB45, MB72 and MB77. The gene, *dfrA30* containing 471 bp long ORF, which shared maximum 93% identical at the amino acid level with the closest known Dfr (*dfrA5*) sequence of *E. coli* (Ac. No. AJ419169) was recognized as a novel gene cassette [12]. The PROSITE motif search has revealed that the dihydrofolate reductase signature sequence, VIGngpdIPWsakg.EqllFkaiT, was intact in most of the Dfr protein sequences.

An array of two cassette genes in class 1 integrons was noted in twenty-seven isolates. The most frequent combination was *dfrA1-aadA1* followed by *dfrA17-aadA5.* The combinations, *dfrA7-aadA5*, *dfrA17-aadA4,* and *dfrA28* -*aadA1* were present in three, two and three isolates respectively. In a two-cassette array detected in MB53, *aadA5* was the second gene after a gene cassette bearing an ORF of 224 amino acids. AadA5 protein from MB53 showed 100% identity with the same protein from a Gram positive bacterium *Staphylococcus epidermidis* (Ac. No. AB291061) and Gram negative bacterium *Enterobacter cloacae* (Ac. No. EF571855). The combination, *dfrA1-orf* (of unknown function) was detected in four isolates, MB38, MB74, MB75 and MB57A. Protein-protein homology of ORF38 derived from MB38 showed that it was 99% identical to the hypothetical protein of *E. coli* (Protein id BAD08521); however, no conserved domain was observed in BlastP analysis. Uniquely represented two-cassettes combinations were *dfr-IIe-arr2* or *oxa1-aadA1*. A combination of three gene cassettes, *dfrA12-orf40A-aadA2*, was detected in MB40A, MB61, and MB67. Three isolates, MB40A, MB61 and MB67, carried two known genes, *dfrA12* and *aadA2*, in addition to a third cassette coding for an unknown protein. Only one isolate, MB35, revealed the array of four gene cassettes, *blaIMP-9-aacA4-oxa10-aadA2*, in its class 1 integron structure. The isolate MB62 and MB63 produced amplicon of size 153 bp, which on analysis revealed that these were empty class 1 integrons devoid of any gene cassette. Several resistance phenotypes were observed with the isolates (MB19, MB20, MB29, MB31, MB41, MB62, MB68, MB76 etc.) for antibiotics like azithromycin, cefipime, cefotaxime, chloramphenicol, ciprofloxacin, levofloxacin, netilmicin, and oxytetracycline which did not correspond to sequences of the gene cassettes amplified from them.

### Test for Association of Two Attributes of the Class 1 Integron Bearing Isolates, Antibiotic Sensitive/resistant Phenotype and Nature of Gene Cassettes of Class 1 Integron (Cassettes Bearing Sequence Homology/No Homology with any known Antibiotic-resistance Gene)

Among 18 antibiotic-sensitive class 1 integron-positive isolates, 14 of them possessed gene cassettes having no homology to any known antibiotic resistance gene and the rest four had antibiotic-resistance gene cassettes. Of the 72 antibiotic resistant class 1 integron-positive isolates, 60 of them possessed antibiotic-resistance gene cassettes and the rest 12 isolates had gene cassettes having no homology to any known antibiotic resistance gene. The observations were classified according to two attributes in a two-way table (*Contingency Table*) having only one degree of freedom [(2−1) × (2−1) = 1]. The null hypothesis is that the two attributes of the class 1 integron-bearing isolates, “antibiotic sensitivity/resistance” and “nature of the gene cassette” are independent. On the hypothesis of independence, the observed value of Yates’ corrected test statistic (χ^2^) 23.28 is greater than the tabulated value (χ^2^ for 1 degree of freedom at the 1% level is 6.63), which is very significant. We therefore reject the null hypothesis at 1% level of significance and conclude that the attributes are not independent; i.e. The data support the alternate hypothesis that ‘class 1 integron bearing antibiotic sensitive isolates have a greater propensity to carry gene cassettes unrelated to known antibiotic-resistance genes’ or in the other way ‘class 1 integron bearing antibiotic resistant isolates have a greater propensity to carry gene cassettes related to known antibiotic-resistance genes’.

### Taxonomic Assignment of Class 1 Integron Positive Isolates

**Figure 3:**
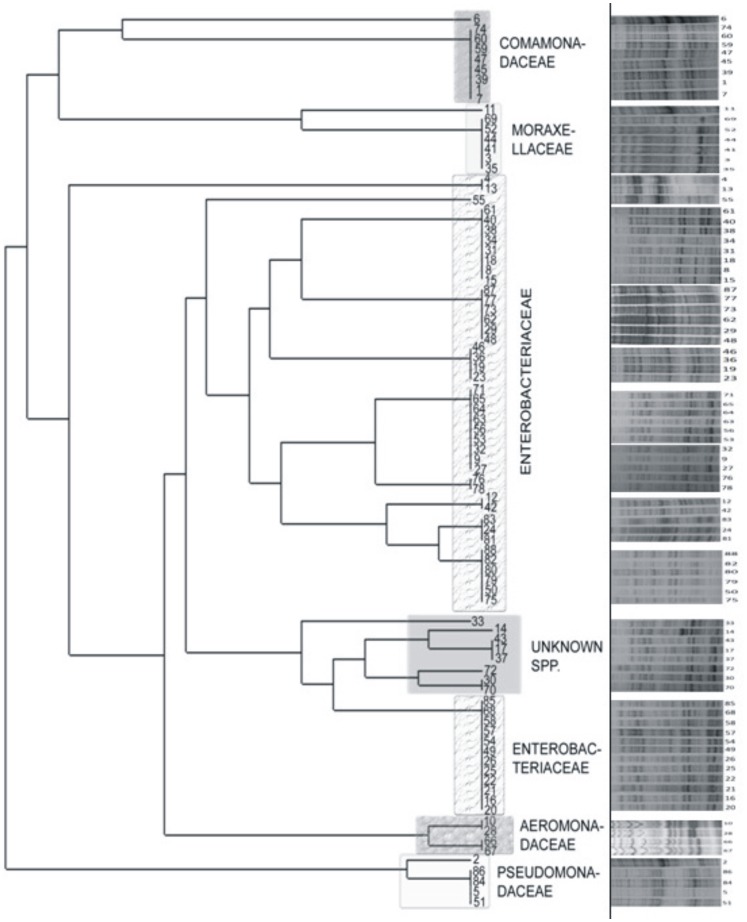
Dendogram based on similarity matrices computed from phenotypic characters and 1D-SDS-PAGE. SDS PAGE anlaysis was done using total protein extracted from bacteria possessing class 1 integrons. MB12 and MB13 were excluded from this analysis. Numbers corresponding to the isolates are: 1, OB 05; 2, OB 12; 3, MB 03; 4, MB 05; 5, MB 08; 6, MB 09; 7, MB 16; 8, MB 19; 9, MB 20; 10, MB 21; 11, MB 22; 12, MB 23; 13, MB 24; 14, MB 25; 15, MB 26; 16, MB 27; 17, MB 28; 18, MB 29; 19, MB 30; 20, MB 31; 21, MB 32; 22, MB 33; 23, MB 34B; 24, MB 35; 25, MB 36; 26, MB 37A; 27, MB 38; 28, MB 39; 29, MB 40; 30, MB 41; 31, MB 42; 32, MB 43; 33, MB 44; 34, MB 45; 35, MB 46; 36, MB 47; 37, MB 48; 38, MB 49; 39, MB 50; 40, MB 51; 41, MB 52; 42, MB 53; 43, MB54; 44, MB 55; 45, MB 56; 46, MB 57; 47, MB 58; 48, MB 59; 49, MB 60; 50, MB 61; 51, MB 62; 52, MB 63; 53, MB 64; 54, MB 65; 55, MB 66; 56, MB 67; 57, MB 68; 58, MB 69; 59, MB 70; 60, MB 71; 61, MB 72; 62, MB 73; 63, MB 74; 64, MB 75; 65, MB 76; 66, MB 77; 67, MB 78; 68, MB 79; 69, MB 80; 70, MB 81; 71, MB 82; 72, MB 83; 73, MR 01; 74, MR 02; 75, MR 03; 76, MR 04; 77, SR 19; 78, NV 66; 79, OD 05; 80, OD 08; 81, OD 10; 82, OC 16; 83, OC 24; 84, OC 74; 85, OC 75; 86, OC 78; 87, OD 21; 88, OD 24.

The biochemical characteristics exhibited by all class 1 integron-positive isolates were compiled (Table S2). Out of 90 integron positive isolates, 89 were Gram-negative, and a single isolate was determined to be Gram-positive. Two isolates, one Gram positive, MB18, and one gram negative, MB12, were excluded from the numerical taxonomy analyses because they responded differently and were not amenable for comparison (of the two, MB18 was assigned to *Brevibacterium siliguirense* sp. nov. by polyphasic approach [13]). All gram negative facultatively oligotrophic isolates were grouped into two major categories, oxidase positive and oxidase negative. All phenotypic characters were converted into binary numbers and similarity matrix was calculated using DendroUPGMA tool. Similarity matrix from phenotypic data was computed (data not shown) and an UPGMA dendogram of all the isolates were constructed (Fig. 3). It was observed that bacteria possessing same phenotype were found to exhibit nearly identical protein profile when grown in same culture conditions.

The 16S rRNA gene sequencing (molecular approach) and phenotypic data (similarity matrix, a numerical taxonomic approach) of representatives of each cluster revealed that all oxidase positive and oxidase negative isolates fell under two main classes, *Betaproteobacteria* and *Gammaproteobacteria* (Table 3). Results revealed that *Betaproteobacteria* comprised of only two genera, *Comamonas* and *Acidovorax* of family *Comamonadaceae* while other isolates were identified under super class *Gammaproteobacteria*. The representative genera of class *Gammaproteobacteria* were constituted of families, *Moraxellaceae*, *Pseudomonadaceae*, *Aeromonadaceae* and *Enterobacteriaceae*. The majority of the identified integration positive oligotrophic bacteria of super class *Gammaproteobacteria* was detected from the family *Enterobacteriaceae*. Nine facultative oligotrophic isolates could not be assigned a specific genus from the data derived either phenotypically or by 16S rRNA gene sequence. The 16S rRNA gene sequences of these unclassified isolates (the isolates which could not be assigned any genus) shared insignificant homologies with the known genera of different classes. The 16S rRNA phylogeny showed that they were branching with uncultured bacteria. However on comparing the phenotype data, the group of unknown species was found to cluster with members of the family Enterobacteriaceae.

**Table 3:** Putatively assigned genera (on the basis of partial 16S rRNA gene sequences) of integron carrying isolates.

S. No	Isolate	Genus	Ac. No.	S. No	Isolate	Genus	Ac. No.
1	^a^OB 05	*Acidovorax* sp.	FM865443	46	MB 57	*Proteus* sp.	f
2	OB 12	*Pseudomonas* sp.	FM865447	47	MB 58	*Acidovorax* sp.	a
3	^b^MB 03	*Acinetobacter* sp.	FM865448	48	MB 59	*Enterobacter* sp.	e
4	^c^MB 05	*Shigella* sp.	FM865446	49	MB 60	*Escherichia* sp.	o
5	^r^MB 08	*Pseudomonas* sp.	FM865444	50	^k^MB 61	*Citrobacter* sp.	HF562228
6	MB 09	*Comamonas* sp.	FM865445	51	MB 62	*Pseudomonas* sp.	r
7	MB 16	*Acidovorax* sp.	a	52	MB 63	*Acinetobacter* sp.	b
8	MB 19	*Klebsiella* sp.	d	53	MB 64	*Salmonella* sp.	g
9	MB 20	*Salmonella* sp.	g	54	MB 65	*Escherichia* sp.	o
10	^p^MB 21	*Aeromonas* sp.	FR677018	55	MB 66	*Kluyvera* sp.	HF562221
11	MB 22	*Acinetobacter* sp.	FR677019	56	MB 67	*Salmonella* sp.	g
12	MB 23	*Serratia* sp.	i	57	MB 68	*Escherichia* sp.	o
13	MB 24	*Shigella* sp.	c	58	MB 69	*Escherichia* sp.	o
14	MB 25	Un. B	l	59	MB 70	*Acidovorax* sp.	a
15	MB 26	*Klebsiella* sp.	d	60	MB 71	*Acidovorax* sp.	a
16	^o^MB 27	*Escherichia* sp.	FN396607	61	MB 72	*Klebsiella* sp.	d
17	^m^MB 28	Un. B	HF562231	62	MB 73	*Enterobacter* sp.	e
18	MB 29	*Klebsiella* sp.	d	63	MB 74	*Salmonella* sp.	g
19	MB 30	*Proteus* sp.	f	64	MB 75	*Salmonella* sp.	g
20	MB 31	*Escherichia* sp.	o	65	MB 76	*Salmonella* sp.	g
21	MB 32	*Escherichia* sp.	o	66	MB 77	*Aeromonas* sp.	q
22	MB 33	*Escherichia* sp.	o	67	^q^MB 78	*Aeromonas* sp.	HF562229
23	^f^MB 34B	*Proteus* sp.	HF562223	68	MB 79	*Escherichia* sp.	o
24	^j^MB 35	*Citrobacter* sp.	HF562227	69	MB 80	*Acinetobacter* sp.	b
25	MB 36	*Escherichia* sp.	o	70	^n^MB 81	Un. B	HF562232
26	MB 37A	*Escherichia* sp.	FN396608	71	MB 82	*Salmonella* sp.	g
27	^g^MB 38	*Salmonella* sp.	HF562224	72	MB 83	Un. B	HF562233
28	MB 39	*Aeromonas* sp.	p	73	MR 01	*Enterobacter* sp.	e
29	^e^MB 40	*Enterobacter* sp.	HF562222	74	MR 02	*Acidovorax* sp.	a
30	MB 41	Un. B	n	75	MR 03	*Citrobacter* sp.	k
31	^d^MB 42	*Klebsiella* sp.	FR677020	76	^h^MR 04	*Providencia* sp.	HF562225
32	MB 43	*Salmonella* sp.	g	77	SR 19	*Enterobacter* sp.	e
33	MB 44	Un. B	HF562230	78	NV 66	*Providencia* sp.	h
34	MB 45	*K. pneumoniae*	FR677021	79	OD 05	*Citrobacter* sp.	k
35	MB 46	*Acinetobacter* sp.	b	80	OD 08	*Citrobacter* sp.	k
36	MB 47	*Proteus* sp.	f	81	OD 10	*Citrobacter* sp.	j
37	MB 48	Un. B	m	82	OC 16	*Citrobacter* sp.	k
38	^d^MB 49	*Klebsiella* sp.	FM865635	83	OC 24	*Citrobacter* sp.	j
39	MB 50	*Acidovorax* sp.	a	84	OC 74	*Pseudomonas* sp.	r
40	MB 51	*Klebsiella* sp.	d	85	OC 75	*Escherichia sp*	o
41	MB 52	*A. johnsonii*	FN263374	86	OC 78	*Pseudomonas* sp.	r
42	^i^MB 53	*Serratia* sp.	HF562226	87	OD 21	*Enterobacter* sp.	e
43	MB 54	Un. B	m	88	OD 24	*Citrobacter* sp.	k
44	MB 55	*Acinetobacter* sp.	b	89	MB 12	Un. B	AM937246
45	MB 56	*Acidovorax* sp.	a	90	MB 18	*B. siliguriense*	AM937247

Un.B, Unclassified bacterium; Isolates exhibiting similar phenotype: a, *Acidovorax* sp.; b, *Acinetobacter* sp.; c, *Shigella* sp.; d, *Klebsiella* sp.; e, *Enterobacter* sp.; f, *Proteus* sp.; g, *Salmonella* sp.; h, *Providencia* sp.; i, *Serratia* sp.; j and k, *Citrobacter*.; l–n, Unknown bacterium; o, *Escherichia* sp.; p and q, *Aeromonas* sp.; r, *Pseudomonas* sp.; *A. johnsonii, Acinetobacter johnsonii*; *B. siliguriense, Brevibacterium siliguirense; K. pneumoniae, Klebsiella pneumoniae*; rDNA, ribosomal deoxyribonucleic acid.

In the present study, 55 integron-positive oligotrophic isolates were identified as the members of the family *Enterobacteriaceae*. Isolates MB05 and MB24 were identified as members of the genus Shigella. Genus *Kluyvera* was only represented by the single isolate MB66. Eight isolates, MB19, MB26, MB29, MB42, MB45, MB49, MB51 and MB72 were recognized as the member of genus *Klebsiella*. Genus *Enterobacter* was represented by six isolates, OD21, SR19, MR01, MB40, MB59 and MB73. Four isolates MB30, MB34, MB47 and MB57 were identified as members of genus *Proteus*. Phenotypic data and 16S rRNA gene sequence homology revealed that isolates MB20, MB38, MB43, MB64, MB67, MB74, MB75, MB76 and MB82 belongs to the genus *Salmonella*. Only two isolates, MR04 and NV66, represented the genus *Providencia*. Genus *Serratia* was represented by isolates MB23 and MB53. Phenotypic and genotypic study revealed that the isolates MR03, MB35, MB61, OC16, OC24, OD05, OD08, OD10 and OD24 represented the genus *Citrobacter*. Twelve isolates, OC75, MB27, MB31, MB32, MB33, MB36, MB37, MB60, MB65, MB68, MB69 and MB79 were tentatively classified under genus *Escherichia*.

Family *Comamonadaceae* was represented by nine isolates, MB09, OB05, MR02, MB16, MB50, MB56, MB58, MB70 and MB71. Isolate MB09 was identified as the member of genus *Comamonas* while rest eight isolates were tentatively characterized under genus *Acidovorax*. *Acinetobacter* was the only genus identified amongst the isolates that fell under the family *Moraxellaceae*. Seven isolates, MB03, MB22, MB46, MB52, MB55, MB63 and MB80 were assigned to the genus *Acinetobacter*. *Aeromonas* was the only genus that was identified for the family *Aeromonadaceae* of super class *Gammaproteobacteria*. Four isolates MB21, MB39, MB77 and MB78 were classified under this genus. Family *Pseudomonadaceae* was represented by a single genus *Pseudomonas*. Five isolates OB12, MB08, MB62, OC74, and OC78 were categorized in the genus *Pseudomonas*.

## Discussion

Bacteria that have an ability to grow in very low nutrient concentration (1.0 to 15 mg of carbon per liter) are termed as oligotrophic bacteria [23]. Oligotrophic bacteria are generally isolated by plating environmental samples on agar –solidified R2A or diluted nutrient/Luria Bertani/Luria broth [4], [5], [7]–[9], [11], [18], [23]–[26]. Unlike obligate oligotrophic bacteria (which are unable to grow in a nutrient-rich medium); facultative oligotrophic bacteria can grow on both nutrient rich and poor media. Occurrences of antibiotic-resistant bacteria in diverse habitats were reported from different parts of the world. Description of genes and biochemical mechanisms that confer resistance to antibiotics was intense over the last five decades. Most of the studies for understanding the phenomenon of antibiotic resistance (the ability of a microorganism to withstand the effects of an antibiotic) are limited to the copiotrophic bacteria. This is because of the availability of two international standard principles, CLSI [27] and EUCAST (http://www.eucast.org/clinical_breakpoints/), for inferring antibiotic susceptibility/resistance of an identified isolate in a typical nutrient-rich Mueller Hunton’s medium. In case of oligotrophic bacteria that grow in nutrient-poor medium, determination of susceptibility/resistance to a specific antibiotic using defined cutoff levels and suitable reference strains was not available. In one of our earlier publications, this bottleneck situation was suitably resolved [11]. Moreover, the genetic elements in which these genes are located were precisely described. Characteristically, these elements are mobile and thus provide multiple opportunities to the resistance genes to spread and disseminate from a bacterium to another. Apart from plasmids and transposons, class 1 integron has been recognized as common agents for the dissemination of antimicrobial resistance genes of diverse microorganisms [28], [29]. Class 1 integrons possess a site-specific recombination system that enables insertion, deletion and reshuffling of discrete genetic devices known as gene cassettes. Gene cassette may be defined as the non-replicating DNA molecule (may exist in free-circular form when not incorporated in an integron) which normally contain only a single gene and an additional short sequence, called a 59 base element that functions as a specific recombination site. The genes carried on gene cassettes usually lack promoters and are expressed from a common promoter present in the 5′-conserved segment of the class 1 integron [30]. Cloning and expression of class 1 integron borne trimethoprim resistance genes from MAR oligotrophic bacteria were reported earlier [11], [12]. The present study is first of its kind to cram diversity of class 1 integron borne gene cassettes in oligotrophic bacteria from river water samples collected from three sampling sites per month for three years in succession.

Randomly selected 2188 test strains represented the facultative oligotrophic bacteria of the River Mahananda. It was observed that 76.2% of the test strains were resistant to at least one of the twelve antibiotics tested. This result was similar to a study conducted with oligotrophic bacteria of the soil where 83.7% of the isolates exhibited antibiotic resistance [16]. Among antibiotic-resistant facultative oligotrophic bacteria, MAR category has surpassed SAR by 6%. Screening of the isolates for the presence of class 1 integron by CS-PCR methodology has revealed that only 4.1% carried class 1 integron. In other studies, class 1 integrons were reported to be present in 3.6% and 3.8% of the total bacteria isolated from an estuarine environment and environments polluted by quaternary ammonia compounds respectively [31], [32]. On statistical analysis it was inferred that incidence of class 1 integron is not equally probable in sensitive, SAR, and MAR oligotrophic bacteria. Sequence analyses of the CS-PCR products have shown that majority of the amplicons >0.7 kb contained gene cassettes, whereas amplicons <0.7 kb were either empty or unrelated to antibiotic resistance (Table 1,2). The common carriages of antibiotic resistance genes were for aminoglycoside-modifying enzymes and trimethoprim-resistant dihydrofolate reductases (DFRs). Two types of DFRs were noted, type I and type II. Genes conferring resistance to β-lactams (e.g. ampicillin, cephalothin, and oxacillin etc.), kanamycin and gentamycin were also found (Table 2). Seven isolates were found to carry two or more gene cassettes in their class 1 integron structure, each cassette coding for antibiotic resistance gene or unknown protein; cassette having ORF for unknown protein was either located in-between the two antibiotic-resistance-gene-cassettes or as the second cassette after the first antibiotic-resistance-gene-cassette in the array (Table 2). Such arrays of gene cassettes in class 1 integrons were reported by earlier authors [33], [34]. The presence of empty (devoid of any gene cassette) class 1 integron was detected in two of the isolates. Empty integron is indicative of an elemental platform for acquiring gene cassettes from the environment.

Gene cassettes coding for proteins other than antibiotic-resistance function were detected in a considerable number of sensitive class 1 integron bearing facultative oligotrophic bacteria of River Mahananda (Table 1). Association of class 1 integron borne gene cassettes coding for proteins other than antibiotic-resistance function with sensitive oligotrophic bacteria was found to be statistically significant. The predicted gene sequences in these cassettes corresponded to ribosomal methyl transferase, ABC transporters, phosphoribosyl formyl glycinamide synthase, hemolysin activator, transposase, pseudouridine synthase, nuclease, helicase etc. (Table 1). Similar gene sequences were reported from culture-independent gene-cassette-metagenome studies [35], [36]. Nonetheless, few reports on gene cassettes unrelated to any antibiotic-resistance function are also available from culturable bacteria [33], [37]. The isolates bearing class 1 integrons fell under two major classes, *Betaproteobacteria* and *Gammaproteobacteria;* the former represented by two genera, *Comamonas* and *Acidovorax* under *Comamonadaceae*, and the latter by the members of the families *Moraxellaceae*, *Pseudomonadaceae*, *Aeromonadaceae* and *Enterobacteriaceae*. The dominance of betaproteobacterial phylotypes and affiliation of 61.76% of betaproteobacterial members to comamonadaceae in the 16S rRNA gene clone library of the metagenome of Mahananda river water was reported earlier [38].

The present study has elucidated the presence of class 1 integrons and also exposed the reservoir of gene cassettes coding for novel genes both related and unrelated to antibiotic –resistance in oligotrophic bacteria.

## Supporting Information

Figure S1 **Experimental design.** Flow diagram describing the sequence of experiments
(DOC)

Figure S2 **Incidence of class 1 integrons in different resistance index groups.**
(TIF)

Table S1 **Resistance phenotype of class 1integron positive oligotrophic bacterial isolates.**
(DOC)

Table S2 **Biochemical characteristics exhibited by class 1 integron bearing facultatively oligotrophic bacteria.**
(DOC)
