# Supplementary material for: Diverse Gene Cassettes in Class 1 Integrons of Facultative Oligotrophic Bacteria of River Mahananda, West Bengal, India
Source: PLoS One. 2013 Aug 9;8(8):e71753. doi: 10.1371/journal.pone.0071753 (PMC3739733; doi:10.1371/journal.pone.0071753)
Supplement: Figure S1 — Experimental design. Flow diagram describing the sequence of experiments (DOC) [file pone.0071753.s001.doc]

Collection of water sample

Dilution series (10^-3^, 10^-5^, 10^-7^, 10^-9^)

Plating of 10^0^, 10^-2^ 10^-3^, 10^-5^, dilution on to 0.001X Luria agar plate containing specific antibiotic

Plating on to 0.001X Luria agar plate containing no antibiotic

Total aerobic colony counting

Numbering (1- n) of discrete colonies developed on 0.001X LA plate without antibiotic followed by generation of 100 ± 10 random numbers by research randomizer; picking up of colonies corresponding to the random numbers to insert in the grids of the master plates ( 1^st^ generation master plate)

Replica plating of 1^st^ Generation Master Plate (s) on to 1X LA and R2A agar plate(s)

Selection of Facultative oligotrophic colonies= √

Obligate oligotroph=X

Replica plating for determination of antibiotic susceptibility profile (Sensitive= susceptible to all 12 antibiotics tested; MAR= resistant to two or more antibiotics tested)

Presence/ absence of Class 1 Integron [all colonies of the 2^nd^ generation master plate]

Detected by PCR assay

Cross checking/ confirmation of antibiotic profile of integron positive isolates

Quantification of total culturable oligotroph resistant to a particular antibiotic (out of 12 different antibiotics used in this study)

Re-numbering of 100 facultative oligotrophic colonies and generation of 25± 2 (n/4) random numbers numbers by research randomizer; picking up of colonies corresponding to the random numbers to insert in the grids of the master plate (2^nd^ generation master plate)

**Figure S1.**
