# Supplementary figures and images for: Diverse Gene Cassettes in Class 1 Integrons of Facultative Oligotrophic Bacteria of River Mahananda, West Bengal, India
Source: PLoS One. 2013 Aug 9;8(8):e71753. doi: 10.1371/journal.pone.0071753 (PMC3739733; doi:10.1371/journal.pone.0071753)

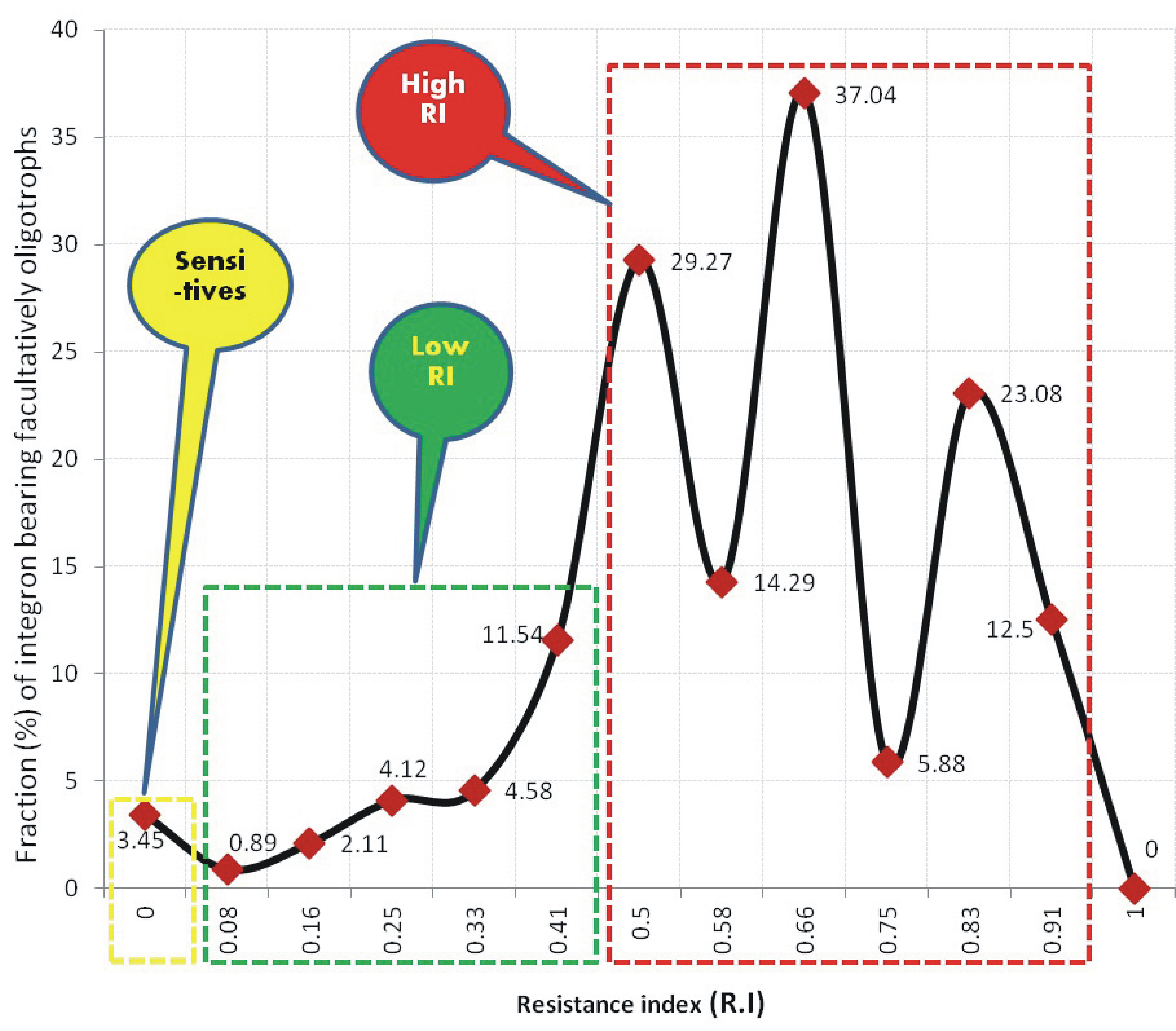

Supplement: Figure S2 — Incidence of class 1 integrons in different resistance index groups. (TIF) [file pone.0071753.s002.tif]
