# Supplementary material for: Diverse Gene Cassettes in Class 1 Integrons of Facultative Oligotrophic Bacteria of River Mahananda, West Bengal, India
Source: PLoS One. 2013 Aug 9;8(8):e71753. doi: 10.1371/journal.pone.0071753 (PMC3739733; doi:10.1371/journal.pone.0071753)
Supplement: Table S1 — Resistance phenotype of class 1integron positive oligotrophic bacterial isolates. (DOC) [file pone.0071753.s003.doc]

**Table S1**

| Group | Resistance index (RI) | Isolate | Phenotype |
| --- | --- | --- | --- |
| 1 | 0.08 | OB 05 | Amp |
|  |  | MB 09 | Tet |
|  |  | MR 01 | Azi |
|  |  | MB 20 | Net |
|  |  | MB 24 | Net |
|  |  | MB 50 | Net |
|  |  | MB 57 | Cot |
| 2 | 0.16 | MR 02 | Cef, Cft |
|  |  | MB 23 | Amp, Str |
|  |  | MB 36 | Str, Tet |
|  |  | MB 49 | Amp, Net |
|  |  | MB 52 | Cot, Str |
|  |  | MB 51 | Amp, Net |
|  |  | MB 64 | Cot, Tet |
| 3 | 0.25 | MB 03 | Amp, Cot, Str |
|  |  | MB 21 | Amp, Chl, Cot |
|  |  | MB 26 | Amp, Cot, Tet |
|  |  | MB 32 | Net, Str, Tet |
|  |  | MB 33 | Net, Str, Tet |
|  |  | MB 42 | Amp, Kan, Tet |
|  |  | MB 67 | Amp, Cot, Tet |
|  |  | MB 77 | Amp, Cot, Tet |
| 4 | 0.33 | MR 03 | Cot, Liv, Net, Tet |
|  |  | MB 47 | Amp, Cot, Net, Tet |
|  |  | MB 62 | Amp, Cft, Chl, Cot |
|  |  | MB 81 | Amp, Cft, Chl, Str |
|  |  | MR 04 | Azi, Lev, Net, Tet |
| 5 | 0.41 | MB 18 | Amp, Cef, Chl, Cot, Net |
|  |  | MB 31 | Amp, Chl, Cot, Str, Tet |
|  |  | MB 40 | Amp, Cft, Cot, Kan, Net |
|  |  | MB 59 | Amp, Cef, Chl, Cot, Net |
|  |  | MB 66 | Amp. Chl, Cot, Str, Tet |
|  |  | MB 72 | Amp, Cef, Cip, Kan, Net |
|  |  | MB 74 | Amp, Cot, Kan, Liv, Tet |
|  |  | MB 75 | Amp, Cot, Kan, Liv, Tet |
|  |  | MB 80 | Cft, Chl, Cot, Str, Tet |
| 6 | 0.50 | MB 27 | Amp, Cef, Cft, Cip, Cot, Tet |
|  |  | MB 30 | Amp, Azi, Cip, Cot, Liv, Tet |
|  |  | MB 41 | Amp, Cft, Chl, Net, Str, Tet |
|  |  | MB 60 | Amp, Cip, Cot, Kan, Net, Tet |
|  |  | MB 61 | Amp, Azi, Cft, Cip, Cot, Net |
|  |  | MB 69 | Amp, Cft, Chl, Cot, Str, Tet |
|  |  | MB 78 | Amp, Cef, Cft, Chl, Cot, Liv |
|  |  | SR 19 | Amp, Cef, Cft, Cot, Str, Tet |
|  |  | OD 05 | Amp, Cft, Cot, Net, Str, Tet |
|  |  | OD 08 | Amp, Cef, Cft, Cot, Str, Tet |
|  |  | OC 16 | Chl, Cot, Liv, Net, Str, Tet |
|  |  | MB 82 | Azi, Cef, Chl, Cip, Kan, Liv |
| 7 | 0.58 | MB 19 | Amp, Cef, Cft, Cip, Cot, Liv, Tet |
|  |  | MB 28 | Amp, Cef, Cft, Cip, Cot, Kan, Net |
|  |  | MB 29 | Amp, Cef, Cip, Cot, Kan,Net, Tet |
|  |  | MB34B | Amp, Azi, Cip, Cot, Liv, Net, Tet |
|  |  | MB37A | Amp, Cef, Cft, Cip, Cot, Net, Tet |
|  |  | MB 53 | Amp, Cef, Cft, Chl, Cip, Cot, Net |
|  |  | MB 70 | Amp, Cef, Cft, Chl, Cot, Kan, Str |
|  |  | MB 73 | Amp, Cef, Cft, Chl, Cip, Net, Tet |
|  |  | OC 78 | Amp, Cef, Cft, Cot, Liv, Net, Tet |
| 8 | 0.66 | MB 25 | Amp, Cef, Cft, Cip, Cot, Liv, Net, Tet |
|  |  | OC 24 | Amp, Cef, Cft, Cip, Cot, Liv, Net, Tet |
|  |  | MB 35 | Amp, Cef, Cft, Cip, Cot, Kan, Net, Tet |
|  |  | MB 45 | Amp, Cef, Cft, Cip, Cot, Net, Str, Tet |
|  |  | MB 79 | Amp, Cft, Chl, Cip, Cot, Liv, Str, Tet |
|  |  | OD 10 | Amp, Azi, Cef, Cft, Cot, Net, Str, Tet |
|  |  | OD 21 | Amp, Azi, Cef, Cft, Chl, Cip, Cot, Lev |
|  |  | OD 24 | Amp, Azi, Cef, Cft, Cip, Cot, Lev, Tet |
|  |  | MB 65 | Amp, Azi, Cef, Cft, Cip Cot, Kan, Net |
|  |  | MB 76 | Amp, Azi, Cef, Cip, Cot, Liv, Str, Tet |
| 9 | 0.75 | OC 74 | Amp, Cef, Cft, Chl, Cip, Cot, Liv, Net, Str |
| 10 | 0.83 | MB 38 | Amp, Cef, Cft, Chl, Cip, Cot, Kan, Liv, Net, Tet |
|  |  | MB 68 | Amp, Azi, Cef, Cft, Chl, Cip, Cot, Liv, Str, Tet |
|  |  | OC 75 | Amp, Azi, Cef, Cft, Chl, Cip, Cot, Liv, Str, Tet |
| 11 | 0.91 | NV 66 | Amp, Azi, Cef, Cft, Chl, Cip, Cot, Lev, Net, Str, Tet |

#### ^a^ number of strains sensitive/or resistant to a particular antibiotics or set of antibiotics.

^b^Amp: ampicillin; Azi: azithromycin; Cef: cefipime; Cft: cefotaxime; Chl: chloramphenicol; Cip: ciprofloxacin; Cot: co-trimoxazole; Kan: kanamycin; Lev: levofloxacin; Net: netilmicin; Str: streptomycin; Tet: oxytetracycline.
