# Supplementary material for: Diverse Gene Cassettes in Class 1 Integrons of Facultative Oligotrophic Bacteria of River Mahananda, West Bengal, India
Source: PLoS One. 2013 Aug 9;8(8):e71753. doi: 10.1371/journal.pone.0071753 (PMC3739733; doi:10.1371/journal.pone.0071753)
Supplement: Table S2 — Biochemical characteristics exhibited by class 1 integron bearing facultatively oligotrophic bacteria. (DOC) [file pone.0071753.s004.doc]

**Table S2**

| Tests | Isolates | | | | | | | | | |
| --- | --- | --- | --- | --- | --- | --- | --- | --- | --- | --- |
|  | **OB05** | **OB12** | **MB03** | **MB05** | **MB08** | **MB09** | **MB16** | **MB18** | **MB19** | **MB20** |
| Indole | - | - | - | + | - | - | - | - | - | + |
| MR | - | - | - | + | - | - | - | - | - | + |
| VP | - | - | - | - | - | - | - | + | + | - |
| Citrate | - | + | + | - | + | - | - | + | + | + |
| Catalase | + | - | + | + | - | - | + | + | + | + |
| Casienase | - | + | - | - | + | - | - | - | - | - |
| Gelatinase | - | - | - | - | - | + | - | + | - | + |
| Amylase | - | - | - | - | + | - | - | - | - | - |
| H2S | - | + | - | - | + | - | - | - | - | - |
| Oxidase | + | + | - | - | + | + | + | - | - | - |
| *Acid from* |  |  |  |  |  |  |  |  |  |  |
| Dextrose | - | - | - | - | - | - | - | - | + | + |
| Dulicitol | - | - | - | + | - | - | - | - | - | - |
| Adonitol | - | + | - | - | + | - | - | - | - | + |
| Cellobiose | - | - | - | - | - | - | - | - | + | - |
| Melibiose | - | - | - | - | - | - | - | - | + | + |
| Mannose | - | + | - | - | + | - | - | + | + | - |
| Trehalose | - | - | - | - | - | - | - | - | + | + |
| Maltose | - | - | - | - | - | - | - | - | + | + |
| Sorbitol | - | + | - | - | + | - | - | - | + | + |

| Tests | Isolates | | | | | | | | | |
| --- | --- | --- | --- | --- | --- | --- | --- | --- | --- | --- |
|  | **MB21** | **MB22** | **MB23** | **MB24** | **MB25** | **MB26** | **MB27** | **MB28** | **MB29** | **MB30** |
| Indole | - | - | - | + | + | - | + | + | - | - |
| MR | + | - | + | + | + | - | + | + | - | - |
| VP | - | - | - | - | - | + | - | - | + | - |
| Citrate | + | + | + | - | - | + | - | - | + | + |
| Catalase | + | + | + | + | - | + | + | + | + | + |
| Casienase | + | - | + | - | - | - | - | - | - | - |
| Gelatinase | + | + | - | - | - | - | + | - | - | + |
| Amylase | + | - | - | - | - | - | - | - | - | - |
| H2S | - | - | - | - | - | - | - | - | - | + |
| Oxidase | + | **-** | **-** | - | **-** | - | **-** | - | - | **-** |
| *Acid from* |  |  |  |  |  |  |  |  |  |  |
| Dextrose | + | - | + | - | + | + | + | + | + | + |
| Dulicitol | - | - | + | + | - | - | + | - | - | - |
| Adonitol | - | - | + | - | - | - | - | - | - | + |
| Cellobiose | - | - | + | - | - | + | - | - | + | + |
| Melibiose | - | - | + | - | + | + | + | + | + | + |
| Mannose | - | - | + | - | + | + | + | + | + | + |
| Trehalose | + | - | + | - | + | + | + | + | + | + |
| Maltose | + | - | + | - | + | + | + | + | + | + |
| Sorbitol | - | - | + | - | + | + | + | + | + | - |

| Tests | Isolates | | | | | | | | | |
| --- | --- | --- | --- | --- | --- | --- | --- | --- | --- | --- |
|  | **MB31** | **MB32** | **MB33** | **MB34** | **MB35** | **MB36** | **MB37** | **MB38** | **MB39** | **MB40** |
| Indole | + | + | + | - | - | + | + | + | - | - |
| MR | + | + | + | - | + | + | + | + | + | - |
| VP | - | - | - | - | - | - | - | - | - | + |
| Citrate | - | - | - | + | + | - | - | + | + | + |
| Catalase | + | + | + | + | + | + | + | + | + | + |
| Casienase | - | - | - | - | - | - | - | - | + | - |
| Gelatinase | + | + | + | + | - | + | + | + | + | - |
| Amylase | - | - | - | - | + | - | - | - | + | + |
| H2S | - | - | - | + | - | - | - | - | - | - |
| Oxidase | **-** | **-** | **-** | **-** | - | **-** | **-** | **-** | + | - |
| *Acid from* |  |  |  |  |  |  |  |  |  |  |
| Dextrose | + | + | + | + | + | + | + | + | + | + |
| Dulicitol | + | + | + | - | + | + | + | - | - | - |
| Adonitol | - | - | - | + | + | - | - | + | - | + |
| Cellobiose | - | - | - | + | + | - | - | - | - | + |
| Melibiose | + | + | + | + | + | + | + | + | - | + |
| Mannose | + | + | + | + | + | + | + | - | - | - |
| Trehalose | + | + | + | + | + | + | + | + | + | + |
| Maltose | + | + | + | + | + | + | + | + | + | + |
| Sorbitol | + | + | + | - | + | + | + | + | - | - |

| Tests | Isolates | | | | | | | | | |
| --- | --- | --- | --- | --- | --- | --- | --- | --- | --- | --- |
|  | **MB41** | **MB42** | **MB43** | **MB44** | **MB45** | **MB46** | **MB47** | **MB48** | **MB49** | **MB50** |
| Indole | + | - | + | - | - | - | - | + | - | - |
| MR | + | - | + | + | - | - | - | + | - | - |
| VP | - | + | - | - | + | - | - | - | + | - |
| Citrate | - | + | + | - | + | + | + | - | + | - |
| Catalase | + | + | + | + | + | + | + | + | + | + |
| Casienase | - | - | - | - | - | - | - | - | - | - |
| Gelatinase | - | - | + | - | - | - | + | - | - | - |
| Amylase | - | - | - | - | - | - | - | - | - | - |
| H2S | - | - | - | - | - | - | + | - | - | - |
| Oxidase | - | - | **-** | - | - | **-** | **-** | **-** | - | **+** |
| *Acid from* |  |  |  |  |  |  |  |  |  |  |
| Dextrose | + | + | + | + | + | - | + | + | + | - |
| Dulicitol | - | - | - | - | - | - | - | - | - | - |
| Adonitol | - | - | + | - | - | - | + | - | - | - |
| Cellobiose | - | + | - | - | + | - | + | - | + | - |
| Melibiose | + | + | + | + | + | - | + | + | + | - |
| Mannose | - | + | - | + | + | - | + | + | + | - |
| Trehalose | + | + | + | + | + | - | + | + | + | - |
| Maltose | + | + | + | - | + | - | + | + | + | - |
| Sorbitol | + | + | + | + | + | - | - | + | + | - |

| Tests | Isolates | | | | | | | | | |
| --- | --- | --- | --- | --- | --- | --- | --- | --- | --- | --- |
|  | **MB51** | **MB52** | **MB53** | **MB54** | **MB55** | **MB56** | **MB57** | **MB58** | **MB59** | **MB60** |
| Indole | - | - | - | + | - | - | - | - | - | + |
| MR | - | - | + | + | - | - | - | - | - | + |
| VP | + | - | - | - | - | - | - | - | + | - |
| Citrate | + | + | + | - | + | - | + | - | + | - |
| Catalase | + | + | + | + | + | + | + | + | + | + |
| Casienase | - | - | + | - | - | - | - | - | - | - |
| Gelatinase | - | - | - | - | - | - | + | - | - | + |
| Amylase | - | - | - | - | - | - | - | - | + | - |
| H2S | - | - | - | - | - | - | + | - | - | - |
| Oxidase | - | - | - | - | - | + | - | + | - | - |
| *Acid from* |  |  |  |  |  |  |  |  |  |  |
| Dextrose | + | - | + | + | - | - | + | - | + | + |
| Dulicitol | - | - | + | - | - | - | - | - | - | + |
| Adonitol | - | - | + | - | - | - | + | - | + | - |
| Cellobiose | + | - | + | - | - | - | + | - | + | - |
| Melibiose | + | - | + | + | - | - | + | - | + | + |
| Mannose | + | - | + | + | - | - | + | - | - | + |
| Trehalose | + | - | + | + | - | - | + | - | + | + |
| Maltose | + | - | + | + | - | - | + | - | + | + |
| Sorbitol | + | - | + | + | - | - | - | - | - | + |

| Tests | Isolates | | | | | | | | | |
| --- | --- | --- | --- | --- | --- | --- | --- | --- | --- | --- |
|  | **MB61** | **MB62** | **MB63** | **MB64** | **MB65** | **MB66** | **MB67** | **MB68** | **MB69** | **MB70** |
| Indole | - | - | - | + | + | + | + | + | + | - |
| MR | + | - | - | + | + | + | + | + | + | - |
| VP | - | - | - | - | - | - | - | - | - | - |
| Citrate | + | + | + | + | - | + | + | - | - | - |
| Catalase | + | - | + | + | + | + | + | + | + | + |
| Casienase | - | + | - | - | - | - | - | - | - | - |
| Gelatinase | - | - | - | + | + | - | + | + | + | - |
| Amylase | + | + | - | - | - | - | - | - | - | - |
| H2S | + | + | - | - | - | - | - | - | - | - |
| Oxidase | - | + | - | - | - | - | - | - | - | + |
| *Acid from* |  |  |  |  |  |  |  |  |  |  |
| Dextrose | + | - | - | + | + | + | + | + | + | - |
| Dulicitol | + | - | - | - | + | + | - | + | + | - |
| Adonitol | + | + | - | + | - | - | + | - | - | - |
| Cellobiose | + | - | - | - | - | + | - | - | - | - |
| Melibiose | + | - | - | + | + | - | + | + | + | - |
| Mannose | + | + | - | - | + | - | - | + | + | - |
| Trehalose | + | - | - | + | + | + | + | + | + | - |
| Maltose | + | - | - | + | + | + | + | + | + | - |
| Sorbitol | + | + | - | + | + | - | + | + | + | - |

| Tests | Isolates | | | | | | | | | |
| --- | --- | --- | --- | --- | --- | --- | --- | --- | --- | --- |
|  | **MB71** | **MB72** | **MB73** | **MB74** | **MB75** | **MB76** | **MB77** | **MB78** | **MB79** | **MB80** |
| Indole | - | - | - | + | + | + | - | - | + | - |
| MR | - | - | - | + | + | + | + | + | + | - |
| VP | - | + | + | - | - | - | - | - | - | - |
| Citrate | - | + | + | + | + | + | + | + | - | + |
| Catalase | + | + | + | + | + | + | + | + | + | + |
| Casienase | - | - | - | - | - | - | + | + | - | - |
| Gelatinase | - | - | - | + | + | + | + | + | + | - |
| Amylase | - | - | + | - | - | - | + | + | - | - |
| H2S | - | - | - | - | - | - | - | - | - | - |
| Oxidase | **+** | - | - | **-** | **-** | **-** | + | + | **-** | **-** |
| *Acid from* |  |  |  |  |  |  |  |  |  |  |
| Dextrose | - | + | + | + | + | + | + | + | + | - |
| Dulicitol | - | - | - | - | - | - | - | - | + | - |
| Adonitol | - | - | + | + | + | + | - | - | - | - |
| Cellobiose | - | + | + | - | - | - | - | - | - | - |
| Melibiose | - | + | + | + | + | + | - | - | + | - |
| Mannose | - | + | - | - | - | - | + | + | + | - |
| Trehalose | - | + | + | + | + | + | + | + | + | - |
| Maltose | - | + | + | + | + | + | + | + | + | - |
| Sorbitol | - | + | - | + | + | + | - | - | + | - |

| Tests | Isolates | | | | | | | | | |
| --- | --- | --- | --- | --- | --- | --- | --- | --- | --- | --- |
|  | **MB81** | **MB82** | **MB83** | **MR01** | **MR02** | **MR03** | **MR04** | **SR19** | **NV66** | **OD05** |
| Indole | + | + | + | - | - | - | - | - | - | - |
| MR | + | + | + | - | - | + | + | - | + | + |
| VP | - | - | - | + | - | - | - | + | - | - |
| Citrate | - | + | - | + | - | + | + | + | + | + |
| Catalase | + | + | + | + | + | + | + | + | + | + |
| Casienase | - | - | - | - | - | - | - | - | - | - |
| Gelatinase | - | + | - | - | - | - | - | - | - | - |
| Amylase | - | - | - | + | - | + | - | + | - | + |
| H2S | - | - | - | - | - | + | - | - | - | + |
| Oxidase | **-** | **-** | **-** | - | **+** | - | - | - | - | - |
| *Acid from* |  |  |  |  |  |  |  |  |  |  |
| Dextrose | + | + | + | + | - | + | + | + | + | + |
| Dulicitol | - | - | - | - | - | + | - | - | - | + |
| Adonitol | - | + | - | + | - | + | + | + | + | + |
| Cellobiose | - | - | - | + | - | + | - | + | - | + |
| Melibiose | + | + | + | + | - | + | + | + | + | + |
| Mannose | - | - | - | - | - | + | - | - | - | + |
| Trehalose | + | + | + | + | - | + | + | + | + | + |
| Maltose | + | + | - | + | - | + | + | + | + | + |
| Sorbitol | + | + | + | - | - | + | + | - | + | + |

| Tests | Isolates | | | | | | | | |
| --- | --- | --- | --- | --- | --- | --- | --- | --- | --- |
|  | **OD08** | **OD10** | **OC16** | **OC24** | **OC74** | **OC75** | **OC78** | **OD21** | **OD24** |
| Indole | - | - | - | - | - | + | - | - | - |
| MR | + | + | + | + | - | + | - | - | + |
| VP | - | - | - | - | - | - | - | + | - |
| Citrate | + | + | + | + | + | - | + | + | + |
| Catalase | + | + | + | + | - | + | - | + | + |
| Casienase | - | - | - | - | + | - | + | - | - |
| Gelatinase | - | - | - | - | - | + | - | - | - |
| Amylase | + | + | + | + | + | - | + | + | + |
| H2S | - | + | - | + | + | - | + | - | + |
| Oxidase | - | - | - | - | **+** | **-** | **+** | - | - |
| *Acid from* |  |  |  |  |  |  |  |  |  |
| Dextrose | + | + | + | + | - | + | - | + | + |
| Dulicitol | + | + | + | + | - | + | - | - | + |
| Adonitol | + | + | + | + | + | - | + | + | + |
| Cellobiose | + | + | + | + | - | - | - | + | + |
| Melibiose | + | + | + | + | - | + | - | + | + |
| Mannose | + | + | + | + | + | + | + | - | + |
| Trehalose | + | + | + | + | - | + | - | + | + |
| Maltose | + | + | + | + | - | + | - | + | + |
| Sorbitol | + | + | + | + | + | + | + | - | + |
